# Conformational Analysis of Misfolded Protein Aggregation by FRET and Live-Cell Imaging Techniques

**DOI:** 10.3390/ijms16036076

**Published:** 2015-03-16

**Authors:** Akira Kitamura, Kazuhiro Nagata, Masataka Kinjo

**Affiliations:** 1 Laboratory of Molecular Cell Dynamics, Faculty of Advanced Life Science, Hokkaido University, Sapporo 001-0021, Japan; E-Mail: kinjo@sci.hokudai.ac.jp; 2 Faculty of Life Sciences, Kyoto Sangyo University, Kyoto 603-8555, Japan; E-Mail: nagata@cc.kyoto-su.ac.jp

**Keywords:** proteostasis, neurodegenerative disease, protein aggregation, protein misfolding, SOD1, FRET, FCS, fluorescent protein

## Abstract

Cellular homeostasis is maintained by several types of protein machinery, including molecular chaperones and proteolysis systems. Dysregulation of the proteome disrupts homeostasis in cells, tissues, and the organism as a whole, and has been hypothesized to cause neurodegenerative disorders, including amyotrophic lateral sclerosis (ALS) and Huntington’s disease (HD). A hallmark of neurodegenerative disorders is formation of ubiquitin-positive inclusion bodies in neurons, suggesting that the aggregation process of misfolded proteins changes during disease progression. Hence, high-throughput determination of soluble oligomers during the aggregation process, as well as the conformation of sequestered proteins in inclusion bodies, is essential for elucidation of physiological regulation mechanism and drug discovery in this field. To elucidate the interaction, accumulation, and conformation of aggregation-prone proteins, *in situ* spectroscopic imaging techniques, such as Förster/fluorescence resonance energy transfer (FRET), fluorescence correlation spectroscopy (FCS), and bimolecular fluorescence complementation (BiFC) have been employed. Here, we summarize recent reports in which these techniques were applied to the analysis of aggregation-prone proteins (in particular their dimerization, interactions, and conformational changes), and describe several fluorescent indicators used for real-time observation of physiological states related to proteostasis.

## 1. Introduction

**Figure 1:**
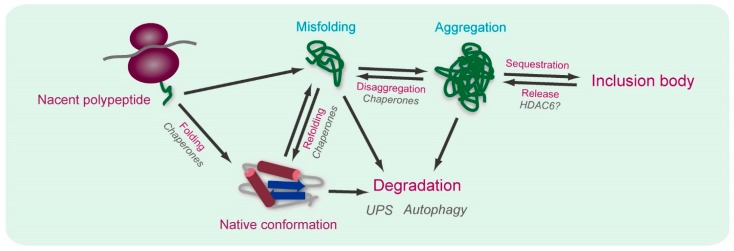
The proteostasis network and the course of aggregation from nascent polypeptide to inclusion body formation.

To maintain cellular homeostasis, it is essential to regulate the quality and amount of each molecule. In particular, homeostasis of the quality and amount of proteins is referred to as proteostasis or proteinstasis [1,2,3,4]. The cellular mechanisms involved in protein quality control (PQC) are comparable to the quality control systems in a factory that makes uniform products. Production of proteins in a cell involves several processes, including transcription, translation, folding, transport, and degradation; the balance between these processes is important to the maintenance of proteostasis. By contrast, disruption of proteostasis affects cell fate, and can result in cell death [5]. One potential cause of such an imbalance is accumulation of misfolded protein. Protein folding is assisted by molecular chaperones, highly abundant molecules that are essential for viability. When misfolded polypeptides form, they are recognized and degraded by proteolysis. Thus, cooperation between molecular chaperones and the protein degradation machinery guards against accumulation of misfolded proteins (Figure 1) [1,2].

Accumulation of misfolded proteins has the potential to disrupt proteostasis. Aging is associated with a continuous imbalance of proteostasis in neurons, resulting in progression of neurodegeneration. Many genes have been implicated in the pathogenesis of various neurodegenerative disorders, including Alzheimer’s disease, Parkinson’s disease, amyotrophic lateral sclerosis (ALS), and Huntington’s disease (HD) [6,7,8]. For example, a missense mutation in superoxide dismutase 1 (SOD1) is causally associated with familial ALS (FALS) [9]. In HD, an expansion of a triplet repeat (CAG) in the huntingtin gene results in production of Huntingtin protein carrying expanded polyQ (Htt-polyQ) [10,11]. Each disease-associated mutant protein is highly aggregation-prone and toxic [2]. A characteristic pathological feature of neurodegenerative diseases is the presence of ubiquitin-positive inclusion bodies containing aggregation-prone proteins in neurons [12,13]. Hence, dysfunctions of the ubiquitin-proteasome system (UPS) and the autophagy–lysosome proteolysis system have been implicated in the pathogenesis of neurodegenerative disorders [14,15].

Studies of proteostasis have employed a large number of methodologies and techniques encompassing molecular biology, cell biology, genetics, physiology, and biochemistry. More recently, however, spectroscopic analyses using fluorescence and luminescence were added to the repertoire [16]. Fluorescence and luminescence are fundamental physical phenomena whose intensities can be measured by spectrometry or microscopy; however, it is difficult to analyze specific protein-of-interest in living cells. How can intensity be modulated to determine the nanostructure and state of specific proteins? To probe the state of specific protein, chemically or genetically labeling to protein-of-interest is an essential technique. Förster/fluorescence resonance energy transfer (FRET) is a mechanism of energy transfer between two fluorophores. Energy of a donor fluorophore in excited state may transfer to an acceptor fluorophore through non-radiative process. Efficiency of FRET effect is affected by the distance and orientation between fluorophores; therefore FRET can be exploited to investigate the structures and interactions of proteins [17]. Fluorescence correlation spectroscopy (FCS) is a system to detect diffusion coefficient and the number of molecules *via* detection of fluctuations in fluorescence intensity caused by the passage of fluorescent molecules through a subfemtoliter detection volume. In low concentration of fluorescent molecule, amplitude of the fluctuation is higher than that in high concentration. When diffusion speed is decreased by increase of molecular mass in solution having same viscosity and temperature, frequency of the fluctuation is decreased. To quantitatively obtain this information in the fluctuation, auto-correlation function (ACF) from the fluctuation is calculated. Half decay time of ACF indicates average residence time of fluorescent molecule in the detection volume; therefore diffusion coefficient of the molecule can be determined from measurement of diffusion coefficient-known fluorescent molecule as a standard. ACF also provides average number of fluorescent molecule in detection volume, thus concentration of fluorescent molecule can be obtained [18,19]. An expanded system of FCS is fluorescence cross-correlation spectroscopy (FCCS), which can determine the number of interacting fluorescent molecules by cross-correlation function between two independent fluorescent fluctuations [20]. Bimolecular fluorescence complementation (BiFC) is a technique to visualize the protein-protein interaction The BiFC is based upon the association of fluorescent protein fragments when two complementary non-fluorescent fragments are brought together by a pair of interacting proteins. Thus, the combination of analytical principles with measurements of fluorescence intensity can provide us with important information about molecules of interest. Here, we review recent studies of proteostasis using spectroscopic imaging methods.

## 2. Spectroscopic Imaging for Elucidation of the Mechanism of Proteostasis 

### 2.1. Detection of Interactions between Aggregation-Prone Proteins and Molecular Chaperones by Förster/Fluorescence Resonance Energy Transfer (FRET) and Fluorescence Correlation Spectroscopy (FCS)

#### 2.1.1. Using FRET to Elucidate the Association between a Chaperone and polyQ Protein

Molecular chaperones, which are conserved in all domains of life, prevent formation of aggregates by misfolded proteins. Fluorescence imaging techniques, such as FRET, were employed to characterize the interactions between aggregation-prone protein and molecular chaperones. For example, the Huntingtin protein is ubiquitously expressed in mammalian cells and tissues; however, Huntingtin that contains an expanded polyQ tract of more than 40 residues (expanded Htt-polyQ) is highly aggregation-prone [21]. One possible source of cellular toxicity in HD is soluble oligomers of expanded Htt-polyQ [22,23,24]. The oligomerization and toxicity of expanded Htt-polyQ is inhibited by molecular chaperones, such as Hsp70–Hsp40, CCT/TRiC, and Prefoldin [22,23,25,26,27,28]. Typically, intermolecular FRET detection of polyQ-protein oligomerization is based on stoichiometric labeling of polyQ-protein with a donor- and acceptor-fluorescent tag and is determined by fluorescence intensity ratio between the donor and acceptor [23,24,29]. After establishing the increase in the FRET efficiency due to oligomerization of expanded Htt-polyQ following incubation and/or agitation of the labeled proteins *in vitro*, one can determine the rate of decrease in the FRET efficiency following the addition of purified molecular chaperones [23]. This rate reveals the kinetics of oligomer elongation of Htt-polyQ in the presence or absence of molecular chaperones. 

#### 2.1.2. Detection of Soluble Oligomers of Expanded polyQ and Mutant Superoxide Dismutase 1 (SOD1) by FCS

Another method for fluorescence detection of soluble oligomers of expanded Htt-polyQ and ALS-linked mutant SOD1 tagged with fluorescent protein is FCS [26,30,31]. FCS can be used for analysis of soluble oligomers in living cells, as well as in solution; however, in conventional systems, it is difficult to quantitatively measure slowly moving or immobile molecules, which typically have diffusion coefficients less than ~0.01 µm^2^/s. Although decrease of diffusion coefficient in living cells indicates increase of molecular mass, it is difficult to conclude existence of homo-oligomers just only by diffusion coefficient. Counts per molecule (CPM) value, which is obtained from the ratio between average fluorescence intensity and the number of molecule in the detection volume on FCS, directly indicates the existence of homo-oligomers of fluorescent protein [31]. Although soluble oligomers in cell lysates can be analyzed by biochemical methods, e.g., sucrose-density gradient and/or gel filtration, followed by Western blotting [25,26,28,31], such biochemical methods are often time-consuming. By contrast, high-throughput and highly reproducible analytical methods like FCS provide fast and reliable detection of soluble oligomers.

#### 2.1.3. Efficient and Reliable Fluorescent Proteins for Use in FLIM-FRET Analysis

One advantage of FRET is that it is independent of the mobility of molecules. To efficiently detect FRET in living cells, fluorescence lifetime imaging microscopy for FRET study (FLIM-FRET) was employed [31,32,33,34]. In FLIM-FRET, proper selection of the fluorescent probe (in particular, the donor) is an essential factor. Use of a fluorescent protein with a single-component fluorescent lifetime (e.g., mTFP1, monomeric teal (cyan) fluorescent protein, or eGFP, enhanced green fluorescent protein) as a donor permits the simplest interpretation of curve-fitting results [35,36]. As acceptors, yellow fluorescent protein (YFP) variants or monomeric orange fluorescent protein (mOrange) have been used with mTFP1 donors [35], whereas tandem dimers or monomers of red fluorescent protein (RFP) variants have been used with eGFP donors [33]. In particular, in two-photon microscopy-based FLIM-FRET, use of a non-fluorescent YFP mutant called REACh (for Resonance Energy-Accepting Chromoprotein) as an acceptor avoids cross-excitation of the acceptor and improves the detection sensitivity of FRET [32,37]. These reliable combinations of fluorescent probes were applied to further analysis of aggregation-prone proteins by FLIM-FRET. Using FLIM-FRET with REACh as the acceptor, a recent study generated a map of interactions between the ALS-linked mutant of SOD1 protein and Hsp70 in the cytoplasm [32]. Moreover, several cyan fluorescent proteins (CFPs) with greatly improved performances and near 4 ns lifetimes were engineered: mTurquoise, mTurquoise2, mCerulean3, and Aquamarine [38,39,40]. These improved CFP variants could be also used for studies of aggregation-prone proteins using FLIM-FRET. In addition to the cytoplasm, molecular chaperones maintain homeostasis in subcellular organelles such as the endoplasmic reticulum (ER), mitochondria, peroxisome, and so on [41,42,43]. A recent study using FLIM-FRET revealed that in the ER, protein disulfide isomerase (PDI) binds calreticulin, a scaffold for glycoproteins, in a manner that depends on the concentration of calcium ion [44]. Notably, FRET analysis can resolve protein interactions in the physiological environment, as opposed to a biochemical lysate.

### 2.2. Characterization of Inclusion Bodies Containing Aggregation-Prone Proteins by FRET

#### 2.2.1. FRET of Sequestered Aggregation-Prone Proteins in Inclusion Bodies

**Figure 2:**
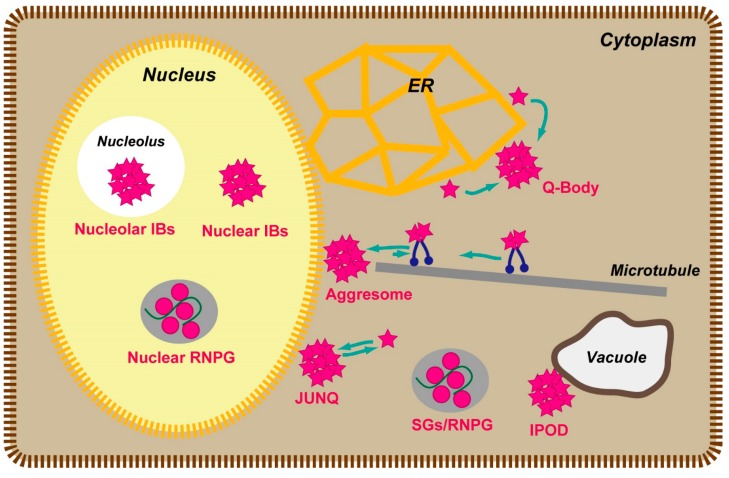
Illustration of typical inclusion bodies (IBs) in eukaryotic cells. Q-body is ER-associated puncta that concentrate different misfolded and stress-denatured proteins en route to degradation. RNPG—ribonucleoprotein granule.

Soluble oligomers in the cytoplasm are gradually sequestered in inclusion bodies [24,31,45]. Several distinct inclusion bodies were identified in the cell: the aggresome, JUNQ (juxtanuclear quality control compartment), IPOD (insoluble protein deposit), and SGs (stress granules) in the cytosol [46,47,48]; the Q-body on the surface of the ER [49]; and nuclear inclusion bodies, nuclear granules, and nucleolar inclusion bodies in the nucleus [50,51,52] (Figure 2). To characterize the conformation of sequestered oligomers in inclusion bodies, FRET analysis of sequestered aggregation-prone proteins in living cells have been employed, using gene-encoded fluorescent protein tags. CFP and YFP, a reliable FRET pair of fluorescent proteins, are a typical choice for studies of inclusion bodies. Htt-polyQ protein tagged with CFP or YFP accumulates and forms inclusion bodies in the cytoplasm [24,53]. FRET efficiency increase with the number of polyQ repeats, and the dynamic properties of the inclusion body decrease as the shorter polyQ tract [53].

#### 2.2.2. Importance of the Orientation Factor in FRET, and Introduction of Circularly Permutated Fluorescent Proteins

**Figure 3:**
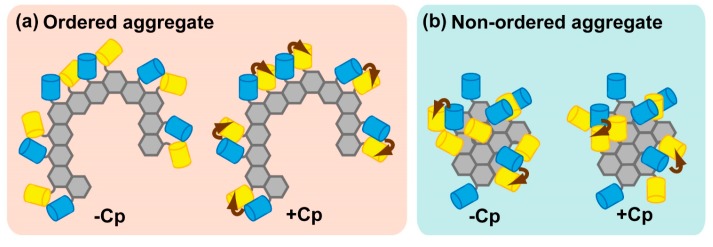
A schematic diagram of modulation of Förster/Fluorescence Resonance Energy Transfer (FRET) efficiency in ordered or non-ordered aggregates using fluorescent proteins. Cyan and yellow barrels show the fluorescent protein as a donor or acceptor, respectively; gray hexagons represent individual units of misfolded proteins; and brown arrows indicate efficient energy transfer. (**a**) In ordered aggregates, introduction of circular permutation (+Cp) dramatically affects FRET efficiency; (**b**) In non-ordered and amorphous aggregates, +Cp barely affects FRET efficiency.

In inclusion bodies in HeLa cells, FRET efficiency of the ALS-linked mutant of SOD1 are low, whereas those of expanded Htt-polyQ are very high [54]. This result suggests that the assembly state of HD-associated expanded Htt-polyQ in cytoplasmic inclusion bodies differs from that of ALS-associated SOD1. When both expanded Htt-polyQ and mutant SOD1 are co-expressed, each protein is sequestered into a distinct compartment [54,55]. The distinct properties of misfolded proteins may result in diversity of pathology. Even if the properties of the ALS-linked mutant of SOD1 differ from those of Htt-polyQ, it is still necessary to elucidate the reason for the low FRET efficiency from inclusion bodies that contain the accumulated protein. In practice, FRET efficiency depends not only on the distance between fluorophores, but also on their relative orientation [56]. To introduce the orientation exchange, circular permutation of fluorescent protein have been employed [57]. Circular permutation is achieved by genetically linking between *N*-terminus and *C*-terminus of fluorescent protein and new *N*- and *C*-terminus that are different from the original position are created without changing the character of fluorescence property. Introduction of cp173Venus, a circular permutation of the YFP variant Venus [57], to the aggregation-prone SOD1 carrying familial ALS-linked glycine 85 to arginine (SOD1-G85R) mutation enables emission of efficient FRET effect when proteasome activity is inhibited; under the same conditions, the use of conventional Venus as an acceptor resulted in no FRET [31]. If oligomers/aggregations of SOD1-G85R form non-ordered amorphous structures, a change in orientation due to circular permutation of the acceptor should not dramatically affect FRET efficiency (Figure 3). These results suggest that SOD1-G85R forms an ordered structure in inclusion bodies when the proteasome is inhibited. In this manner, introduction of circular permutation (in particular, using fluorescent proteins) is an important means for discovering efficient FRET conditions. Although Thioflavin T (ThT) staining can reveal whether an ordered structure is amyloid, it also increases its fluorescence upon binding to beta-sheet rich peptides [58]; consequently, it is difficult to quantitatively distinguish amyloid or beta-sheet structure solely by fluorescence intensity. Hence, FRET measurements in combination with modulation of orientation can reveal the assembly states of sequestered aggregation-prone proteins in inclusion bodies.

#### 2.2.3. Physiological Relevance of Ordered Sequestration of Mutant SOD1 in Inclusion Bodies

Why does mutant SOD1 in inclusion bodies form an ordered structure? Intriguingly, sequestered SOD1 in the inclusion body is released into the cytoplasm during recovery of proteasome activity [31]. If the inclusion body is formed by amorphous and randomly assembled SOD1, this release step may be a thermodynamically unfavorable reaction because refolding process is essential to eliminate entwined polypeptides from inclusion compared with release of a unit of misfolded protein. Release is likely to result in efficient degradation of sequestered SOD1. Moreover, during release of mutant SOD1, the FRET efficiency of SOD1 remaining in the inclusion body gradually weakens, suggesting that the conformation and/or assembly state of mutant SOD1 may change slowly during the release process [31]. On the other hand, HDAC6 is required for release of poly-ubiquitinated proteins in the inclusion body during recovery of proteasome activity [59]. HDAC6 is a histone deacetylase (HDAC) that localizes around inclusion bodies in the cytoplasm [60,61]. Almost all HDACs deacetylate both histones and other proteins to regulate epigenetic processes [62]. Furthermore, cytotoxicity by the ALS-linked mutant SOD1 is elevated during recovery of proteasome activity [31]. Hence, inclusion bodies formed by proteasome inhibition may be quality control compartments that play protective roles by preventing increases in the concentrations of misfolded proteins in the cytoplasm.

### 2.3. Dimerization Detection of ALS-Associated SOD1 Protein

SOD1 dimerization is essential for the enzymatic activity of superoxide dismutase [63,64,65]. Loss of SOD1 dimerization leads to misfolding and changes the course of aggregation [66]. In the absence of proteasome inhibition, dimerization between wild-type SOD1 tagged with donor- and acceptor-fluorescent protein at *C*-terminus, SOD1-mTFP1 and SOD1-Venus/cp173Venus, respectively, cannot be detected by FLIM-FRET analysis [31], whereas dimerization between SOD1-tagged with CFP at *N*-terminus (CFP-SOD1) and SOD1 tagged with YFP at *C*-terminus (SOD1-YFP) can be clearly detected [67]. This difference reveals the importance of the position of the fluorescent protein tag, which is in turn a reflection of the importance of the relative orientation between fluorophores. Furthermore, dimerization of SOD1 can be observed by BiFC (Bimolecular fluorescence complementation) [68], a reconstruction-dependent method for dimerization detection. Dynamic dimerization of SOD1 in living cells is determined by BiFC [67]. Thus, a combination of BiFC and FRET can simultaneously detect the interacting partner of a dimeric/oligomeric protein. It is necessary to perform appropriate investigations to construct an efficient FRET system due to restrictions on structural orientation; nonetheless, FRET provides crucial information regarding the structure of aggregation-prone proteins even if their structures have not been determined by X-ray crystallography or NMR structural analysis. Moreover, FRET and BiFC can be used to explore changes in the orientations and conformations of proteins, in addition to their dimerization, in living cells.

### 2.4. Biosensors to Monitor Physiological Reactions and States Related to Proteostasis in Cells and Tissues

#### 2.4.1. Indicator for Real-Time Detection of Apoptosis by FRET or FCCS

**Figure 4:**
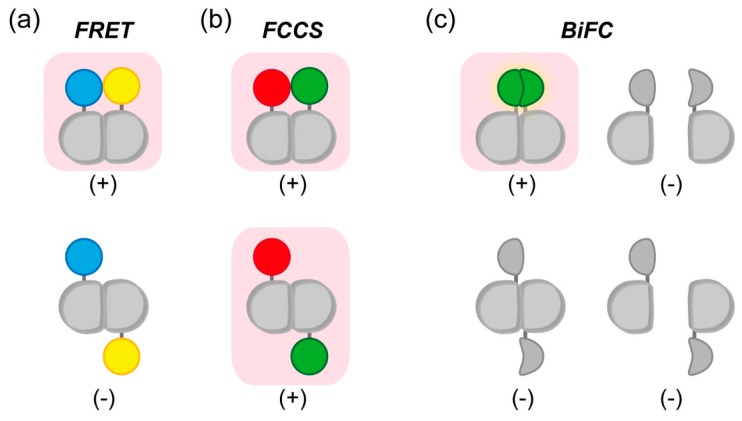
Detection of orientation-dependence or -independence of dimer formation by FRET, FCCS, or BiFC. Magenta background color and (+)/(−) indicates signal emission in each methodology; (**a**) Restriction of FRET effect in dimerization depending on the tagging position of fluorescent molecule. Blue and yellow colors indicate FRET donor and acceptor, respectively; (**b**) Despite the tagging positions of the fluorophores, FCCS can detect dimerization. Green and red colors indicate fluorescence tag; (**c**) In addition to monomeric state (right top and bottom) with no fluorescence, not-appropriate tagging position of the fragments also shows no fluorescence (left bottom). In BiFC, as in FRET, only the appropriate positioning of the protein domains results in fluorescence (green color, left top).

Detection of cell death is an important method for the experimental study of the pathophysiology of neurodegenerative disorders. Apoptosis is a major pathway of programmed cell death. During activation of apoptosis pathway in the cell, caspases, a family of cysteine proteases, are activated and digests substrate sequence in proteins; e.g., other downstream caspases, nuclear lamins, and PARP (poly-ADP ribose polymerase). Although the role of substrate cleavage by caspase remains unclear, protease activity of caspase is essential for progression of apoptosis. To detect apoptosis in living cells, several indicators based on the FRET principle were established. Almost all apoptosis indicators are based on the disappearance of FRET efficiency following cleavage of a specific caspase-recognizing linker peptide between the donor and acceptor fluorophores. Several FRET-based indicators for caspase 3 activation use the linking DEVD peptide, a caspase 3 substrate [69,70,71,72]; one such indicator is SCAT3.1 [73], in which the DEVD sequence is inserted between CFP and Venus. Before caspase 3 activation, SCAT3.1 emits FRET efficiency; activated caspase 3 cleaves the DEVD sequence, resulting in loss of the FRET efficiency. Accordingly, a red-shifted indicator for caspase 3 (LSSmOrange-DEVD-mKate2) was established to allow simultaneous imaging with CFP–YFP [74,75]. Moreover, a FCCS-based caspase 3 indicator, GFP-DEVD-RFP, was also established [76]. FCCS, which represents an expansion of FCS, directly detects the number of interacting fluorescent molecule; thus cleavage between GFP and RFP can be measured as decrease of the number of interacting molecules. One benefit of FCCS is that there is no restriction on the orientation between fluorophores, which is not the case when constructing FRET-based indicators; consequently, however, FCCS has limited application to mobile molecules because of detection of fluorescence fluctuation (Figure 4).

#### 2.4.2. Direct Measurements of Dissociation Constants in Living Cells

Disaggregation of accumulated misfolded protein is a primitive reaction that cooperates with proteolysis to maintain cellular homeostasis. The interaction between Sup35, a yeast prion protein [77,78], and Hsp104, a disaggregase for the prion protein, can be detected by FCCS [79]. In general, FCCS analysis can determine both the concentration of two types of fluorescent molecules and the concentration of interacting molecules, allowing direct measurements of the dissociation constant (*K*_d_) in living cells [80,81,82]. Although FCCS is the only method for directly evaluating *K*_d_ in living cells, the values obtained should be carefully interpreted due to competition with endogenous non-fluorescent protein. To reduce or remove the influence of such competition, it is desirable to perform knockdown or knockout of the endogenous gene of interest.

#### 2.4.3. Calcium and Redox Sensors 

Cellular physiology also plays an important role in the maintenance of proteostasis. The ER maintains both calcium and redox homeostasis [83,84]. To detect changes in the concentration or flux of calcium ion, a large number of indicators and biosensors were established. Two types of fluorescent protein-based calcium ion biosensors have been engineered; one is that FRET donor- and acceptor-fluorescent proteins are fused to the sensory domain, in which conformation by interaction with calcium ion is closed. Another one is that beta-barrel structure near chromophore of fluorescent protein is distorted by conformational change of a fused sensory domain by binding to calcium ion, therefore fluorescence intensity is ratiometrically changed depending on the concentration of calcium ion [83,85]. It is not easy to target chemical indicators in the ER; therefore, genetically encoded biosensors are employed for measurements in the ER (e.g., Pericams, Cameleons, and GCaMP) [85]. For a similar reason, genetically encoded biosensors are also utilized to detect the redox state in the ER. Redox homeostasis in the ER is essential for maintenance of disulfide bond formation; many secretory proteins that pass through the ER include several disulfide bonds, which help the proteins maintain rigid structures in the extracellular environment. A key molecule counteracting redox imbalance is glutathione (GSH). The cysteine side chain in GSH provides redox equivalents; oxidation of GSH results in generation of a dimer, GSSG. Within the cell, the GSH/GSSG balance governs redox homeostasis. Disruption of the GSH/GSSG balance leads to protein misfolding, including formation of mixed disulfide bonds, in the ER. Examinations of redox state in the ER were facilitated by genetically encoded fluorescent redox sensors, such as roGFP, roYFP, Hyper, and Redoxfluor, in which the fluorescence spectrum is changed by redox state of a responsible motif, including two cystein residues near fluorophore of GFP; therefore, ratiometric measurement of fluorescent intensity provides the recovery of redox state after addition of reductant or oxidant [86,87]. In addition, a recently published blue-shifted redox sensor called Oba-Q derived from CFP and Sirius, an ultramarine fluorescent protein [88], is available for use in conjunction with other fluorescent sensors [89]. Recently, expression of a modified form of a cytosolic GSH-degrading enzyme, ChaC1, in the ER lumen slowly recovered redox state after a brief reductive pulse by using roGFP, however, the depletion of GSH had no effect on disulfide-dependent misfolded proteins [90]. This report suggests the existence of alternative protein thiol reductants in the ER. Almost all physiological studies to date have examined changes in biosensors during folding stress induced by dithiothreitol or hydroxyl peroxide. To examine physiological non-equilibrium redox states, not only in the ER but also in other subcellular environments, new biosensors with high signal-to-noise ratios and response speeds will be required.

#### 2.4.4. Fluorescent or Luminescent Reporters for Misfolding and Stress Responses.

Typically, efforts to improve fluorescent proteins and luciferases focus on rapid maturation speed and efficient folding. From another perspective, however, less efficiently folding and more chaperone-dependent proteins (*i.e*., misfolding reporters) can be used as sensors of the proteostasis state due to visualize dysregulation state of folding activity in living cells. For example, in mammalian cells, thermally unstable mutants of American firefly (*Photinus pyralis*) luciferase (FLucSM/FLucDM) form aggregates upon depletion of chaperones or expression of expanded Htt-polyQ [91]. In muscle or neuron in *C. elegans*, FLucSM/FLucDM accumulates following heat shock stress or as a result of aging [91]. In addition, misfolded YFPs (YFPm1 to m4), which emit very little fluorescence and partition into the insoluble fraction, are available as inducers of stress responses [92]. A possible application of these reporters is to discover novel quality control compartments in which misfolded aggregation-prone protein is accumulated in living cells.

## 3. Conclusions 

Fluorescence imaging techniques provide useful and reliable methods for clarifying the mechanism by which protein aggregation dysregulates proteostasis. In particular, FRET and FCS/FCCS can be used for detection of oligomerization and aggregation. The course of aggregation of ALS-linked SOD1 was revealed by FRET and FCS [31,67]. Furthermore, the structure and dynamics of SOD1 in inclusion bodies are different from those of Htt-polyQ [26,31,54,93]. A restricted point of FCS to validate protein interaction is not sensitive to increase of molecular mass. To overcome the sensitivity, FCCS is available and can directly detect protein-protein interaction. Due to differences in the underlying principles, FRET and FCCS are complementary techniques. In FRET, exchange of the orientation factor by circular permutation of fluorescent proteins provides information about the assembly state of proteins, but the size of the molecules remains unclear. By contrast, FCCS measurements can be used to reveal the interaction strength (e.g., *K*_d_) and size of fluorescence-tagged molecules; however, because there is no restriction on the orientation between fluorophores, this method is not sensitive to structural differences. Thus, FRET is a powerful tool for determining conformational changes of proteins. BiFC have a merit to turn off the existence of not interacting protein, however, appropriate orientation of the fragments between interacting proteins is essential as well as FRET. FRET, FCS/FCCS, and BiFC with improved biosensors are available for studies of the biophysical properties of aggregation-prone proteins, as well as for drug screens aimed at identifying molecules that inhibit oligomerization and aggregation; the resultant drugs could be used to slow the progression of neurodegenerative disorders.
